# A Recurrent Increase of Synchronization in the EEG Continues from Waking throughout NREM and REM Sleep

**DOI:** 10.1155/2014/756952

**Published:** 2014-02-06

**Authors:** Ralf Landwehr, Andreas Volpert, Ahmad Jowaed

**Affiliations:** ^1^Klinik für Neurologie, Pfalzklinikum für Neurologie und Psychiatrie, Weinstraße 100, 76889 Klingenmünster, Germany; ^2^Marienkrankenhaus St. Wendel, Stroke Unit, 66606 St. Wendel, Germany; ^3^Klinik für Neurologie, Westpfalz Klinikum GmbH, Hellmut-Hartert-Straße 1, 67655 Kaiserslautern, Germany

## Abstract

Pointwise transinformation (PTI) provides a quantitative nonlinear approach to spatiotemporal synchronization patterns of the rhythms of coupled cortical oscillators. We applied PTI to the waking and sleep EEGs of 21 healthy sleepers; we calculated the mean levels and distances of synchronized episodes and estimated the dominant frequency shift from unsynchronized to synchronized EEG segments by spectral analysis. Recurrent EEG synchronization appeared and ceased abruptly in the anterior, central, and temporal derivations; in the posterior derivations it appeared more fluctuating. This temporal dynamics of synchronization remained stable throughout all states of vigilance, while the dominant frequencies of synchronized phases changed markedly. Mean synchronization had high frontal and occipital levels and low central and midtemporal levels. Thus, a fundamental coupling pattern with recurrent increases of synchronization in the EEG (“RISE”) seems to exist during the brain's resting state. The generators of RISE could be coupled corticocortical neuronal assemblies which might be modulated by subcortical structures. RISE designates the recurrence of transiently synchronized cortical microstates that are independent of specific EEG waves, the spectral content of the EEG, and especially the current state of vigilance. Therefore, it might be suited for EEG analysis in clinical situations without stable vigilance.

## 1. Introduction

The EEG frequency spectrum and the distribution of EEG transients vary extensively across the wake-sleep cycle. Since most EEG waves are synchronized across several EEG leads, an expansion of the conventional single-channel evaluation to regional and global EEG coupling analysis can reveal complex intracortical interactions. Coherence and cross correlation and other linear measures have been applied effectively to the EEG [1–7]. However, algorithms that comprise nonlinear as well as linear interactions on short time scales (within the millisecond range) often permit a more precise estimation of the dynamics of spatiotemporal EEG synchronization. Nonlinear measures like nonlinear interdependences and phase synchronization can detect interhemispheric synchronization with higher sensitivity than cross-correlation and coherence function [8]. Like the conventional EEG parameters, these linear and nonlinear synchronization measures seem to be subject to similar changes across the wake-sleep cycle: the amount, the spatial distribution, and the temporal dynamics of EEG synchronization depend on the respective waveforms as well as on the current state of vigilance and alertness [2, 5, 9–13].

In contrast, in a former study of EEG synchronization during REM sleep [14], we found evidence that at least a portion of the cortical synchronization seems to be independent of the current state of vigilance. In a previous study, the coupling dynamics of EEG activation phases in NREM sleep was characterized by marked recurrent increases of coupling during EEG activation phases in the central leads [15]. We also detected a comparable recurrent increase of synchronization in the REM sleep EEG [14]. These findings suggest the possible existence of a spatially and temporally stable pattern of EEG synchronization across all levels of vigilance during the sleep-wake cycle.

Pointwise transinformation (PTI) provides an algorithm to detect and quantify dynamic coupling processes in the EEG, capturing both linear and nonlinear interactions [16–19]. An outstanding feature of the PTI as a measure of EEG synchrony is the combination of a high temporal resolution with a clear separation of high and low synchronization phases [14]. PTI can be easily interpreted as the mutual coupling of pairs of EEG leads; it might also provide indirect information about the synchronization of neuronal assemblies in the corresponding cortical areas [19, 20].

The objective of the present study was the identification and quantification of synchronization phases in the waking and NREM and REM sleep EEG using the PTI algorithm. Based on our previous studies, we expected to find a recurrent pattern of synchronization independent of both specific EEG waves and graphoelements (like *K*-complexes, sleep spindles, and delta waves) and the spectral content of the EEG. If this pattern corresponds to a physiological mechanism which synchronizes different sites of cortical and probably also subcortical networks, the distributions of the durations of synchronized and unsynchronized EEG sections assessed by PTI should be consistent for all waveforms and states of vigilance. Taking into account the results of other EEG synchronization studies, this would imply that the mean level and the dynamic changes of cortical synchronization depend only partially on the current state of vigilance, while other synchronization patterns are characterized by a constant spatiotemporal distribution; the latter patterns should be detected by PTI. In addition, clinical situations with an instable vigilance are common. In these situations, conventional EEG parameters are subject to marked changes; in contrast, PTI as an EEG synchronization measure that is largely independent of vigilance could provide a valuable approach to measuring fundamental cortical coupling patterns.

## 2. Materials and Methods

### 2.1. Participants

A database of all adults who had been admitted to the neurological clinic for health checks and had been reported to be healthy sleepers was used to identify eligible participants. We evaluated retrospectively the data of 21 adults—12 women and 9 men aged 18.3–47.2 years. Sleep-wake disorders were excluded by medical history, pupillographic sleepiness test, and sleep questionnaires (Pittsburgh Sleep Quality Index, Epworth Sleepiness Scale). Exclusion criteria were the intake of substances with central nervous effects, a diagnosis of concomitant or former neurological or psychiatric disorders, and abnormal neurological, medical, or psychiatric status. All participants had given informed consent for their EEG data to be used for further evaluation.

### 2.2. EEG Derivations

A waking EEG and a sleep EEG of each participant were evaluated. The waking EEGs had been derived in the morning after undisturbed sleep (with 21 leads according to the 10–20 system: FP1, FP2, F3, Fz, F4, C3, Cz, C4, P3, Pz, P4, O1, O2, F7, F8, T3, T4, T5, T6, A1, and A2; common average reference) following standard procedure (20 minutes; TC, 0.3 s; HF, 70 Hz; sampling rate, 250 Hz; Ag-Ag-Cl electrodes). The sleep EEGs had been derived for a complete night of normal sleep in a laboratory for video-EEG polygraphy with 18 leads (FP1, FP2, F3, F4, C3, C4, P3, P4, O1, O2, F7, F8, T3, T4, T5, T6, A1, and A2; common average reference) and the same technical parameters. Sleep EEGs with pathological sleep profiles were excluded. All EEG derivations included electromyography (EMG) of the mental muscles, electrooculogram (EOG), and ECG by a D2 lead. The EEGs were recorded with a Schwarzer Brainlab 3.30-0.00 system and stored on hard disc. Visual sleep scoring [21] by two sleep specialists was based on 30 s epochs of C3 and C4 leads with F3/F4 and O1/O2 as auxiliary channels (common reference). Epochs with visually recognizable EEG artifacts were excluded from further evaluation.

### 2.3. EEG Analysis

Details of the PTI algorithm are given in the appendix. PTI was calculated for all EEGs with segments of 60 s duration (15000 data points) for FP1-FP2, F3-F4, C3-C4, P3-P4, O1-O2, F7-F8, T3-T4, T5-T6, A1-A2, F3-O1, and F4-O2. Since circadian changes of the waking EEG have been described [22], the waking EEGs were evaluated separately (“W_day_”) from the waking phases in the sleep EEGs (“W_night_”). Mean PTI, mean duration of elevated synchronization phases, and mean duration of unsynchronized intervals were assessed separately for each state of vigilance (W_day_, W_night_, N1, N2, N3, and REM). Elevated synchronization phases were defined as phases with a PTI level ≥ mean PTI + 1 standard deviation. Since absolute values of PTI depend on the selection of the embedding parameters and only relative changes of PTI were of concern for our study, PTI was normalized to 1 by maximal/minimal values.

2 s intervals around all synchronized episodes were also analyzed by an FFT spectral analysis with a frequency range of 0–25 Hz and a frequency resolution of 0.5 Hz. The frequency bands were defined as delta 0.5–3.5 Hz, theta > 3.5–8 Hz, alpha > 8–12 Hz, sigma > 12–14.5 Hz, and beta > 14.5–25 Hz. The power spectrum of the 2 s interval beginning with the onset of the elevated coupling was compared to the previous unsynchronized 2 s interval. The dominant frequency shift was then defined by the frequency band with the highest increase of relative power.

### 2.4. Statistics

All EEG epochs without visually identified artifacts were analyzed. The distribution of all PTI parameters could be approximated by a lognormal distribution, which we confirmed by a Kolmogorow-Smirnow test. Stage dependent differences of the PTI parameters were assessed for all patients by an analysis of variance after a logarithmic transformation. Significance was assumed for *P* < 0.05. Since 3 parameters for 11 pairs of EEG leads and 6 states of vigilance were compared, an *α*-adjustment for multiple comparisons was made. The false discovery rate was controlled by the Benjamini-Hochberg procedure [23]: when *m* hypotheses are tested, the *P* values are ordered as *p*_1_ ≤ *p*_2_ ≤ ⋯≤*p*_*m*_, and *k* : = max {*i* : *p*_*i*_ ≤ *q* · *i*/*m*} is defined with *q* = the control level of the false discovery rate. The hypotheses *H*_1_^0^,…, *H*_*k*_^0^ are then rejected. All calculations were performed by a PC-based statistical program (MacAnova 5.05, release 3, University of Minnesota, 2006).

## 3. Results

**Figure 1:**
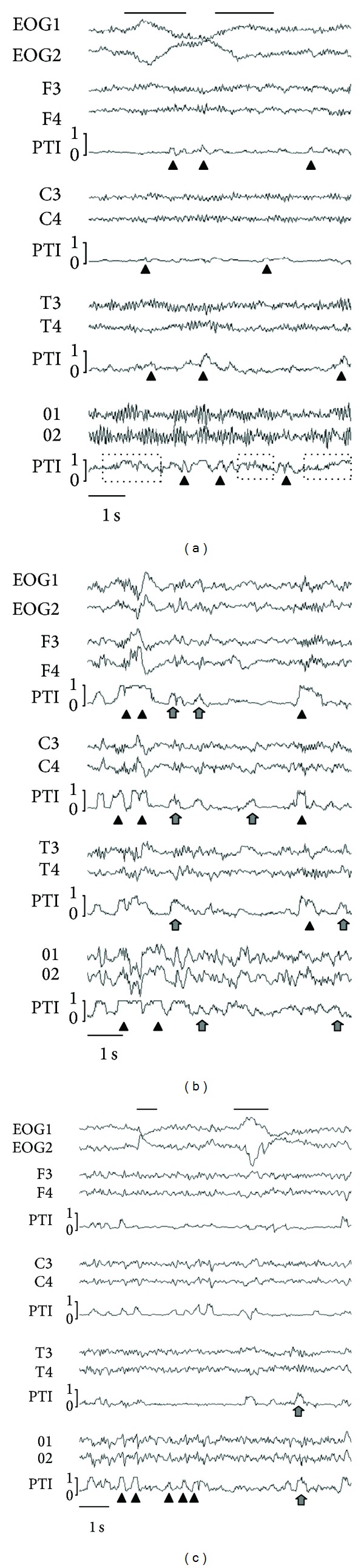
EEG and pairwise pointwise transinformation (PTI) of frontal, central, midtemporal, and occipital derivations. Left and right EOG are displayed additionally. The rapid changes of PTI are visible in the respective regions and different states of vigilance. Note that some increases of PTI are clearly associated with distinct EEG waves (e.g., alpha waves, *K*-complexes, and sleep spindles), while for others, this association is not evident. (a) Waking phase during the night. The rather abrupt changes of PTI with burst-like appearances of synchronization in all derivations (black triangles) and the sometimes waxing and waning characteristics of the occipital synchronization (dotted boxes) are visible. The slow eye movements in the EOG (black bars) have no impact on frontal PTI. (b) NREM sleep stage N2. The burst-like dynamics of PTI is even more evident in the frontal and central derivations than in waking. Synchronization peaks with not only *K*-complexes and sleep spindles (black triangles), but also many other EEG elements like theta waves and less distinct events (arrows). The large potential in the EOG is a *K*-complex, not a REM. (c) REM sleep. As expected, sawtooth waves (black triangles) and alpha waves (arrows) appear highly synchronized. Rapid eye movements (black bars) do not cause increases of frontal synchronization.

**Figure 2:**
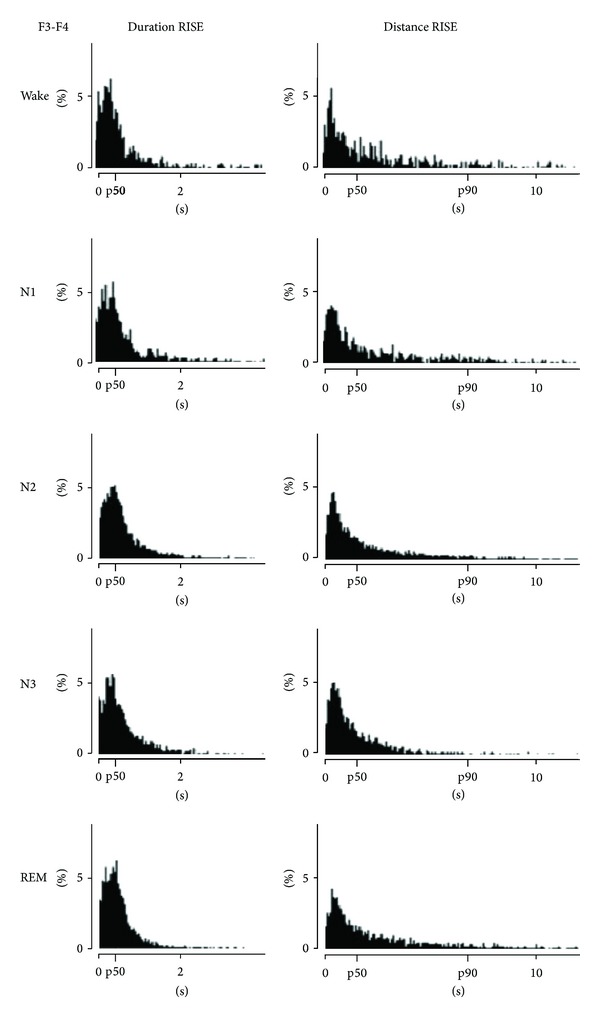
Distributions of the durations of RISE episodes and the distances between successive RISE episodes for waking and all sleep stages, F3-F4. Results for one patient. The distributions can be approximated by a log-normal distribution. The median duration (p50) of synchronization phases is 350–450 ms and does not change with the state of vigilance. A shorter duration of the intervals separating elevated synchronization phases (ca. 1000 ms) is visible in N3 versus W, N1, N2, and R (1900–2200 ms). The 90% percentile (p90) is clearly reduced in N3 compared to the other stages, indicating a predominant reduction of long unsynchronized phases. This indicates a methodological rather than a physiological effect (see Section 4).

All persons had physiological sleep profiles with normal sleep duration, latencies, and percentages of sleep stages (Table 1). A consistent synchronization pattern was found for all states of vigilance, with recurrent increases of PTI (Figure 1); this consistency was verified for intra- and interindividual distributions of PTI throughout the single EEG recordings and across all derivations, respectively. The qualitative distribution of the PTI parameters did not change during the night across subsequent sleep cycles or for any sleep stage (Figure 2). However, the modulation of this pattern differed regionally: in the frontal, temporal, and central leads, burst-like short phases of elevated synchronization were separated by longer unsynchronized intervals, while in the parietal and occipital leads, more frequent and gradually fluctuating synchronization was present (Figure 1). Left- and right-hemispheric synchronization (F3-O1 and F4-O2) had similar characteristics as the frontal synchronization F3-F4 without hemispheric differences. Since PTI parameters of the waking phases during the day (W_day_) and the night (W_night_) did not differ, these phases were summarized for all further evaluations and referred to as stage W.

The mean PTI levels and durations of synchronization phases varied only subtly across the different states of vigilance (Figure 2); significant differences were found only for the mean PTI level of N3 versus W, N1, and R in F7-F8 and T3-T4 and for N3 versus R also in F3-F4, T5-T6, and A1-A2 (Table 2). The median duration of synchronization phases was ca. 350–450 ms. The intervals separating elevated synchronization phases were 1900–2200 ms in W, N1, N2, and R. In contrast, a significantly shorter duration of ca. 1000 ms was distinguished in N3 for the frontal, anterior temporal, midtemporal, and temporobasal leads (*P* < 0.05). This difference was especially prominent for the longest intervals, distinguishable by a significantly reduced p90 value (*P* < 0.05) for these derivations (Figure 2). However, these differences between N3 and the other stages were removed by division by the relative amount of delta in the respective EEG channels (see Section 4).

**Figure 3:**
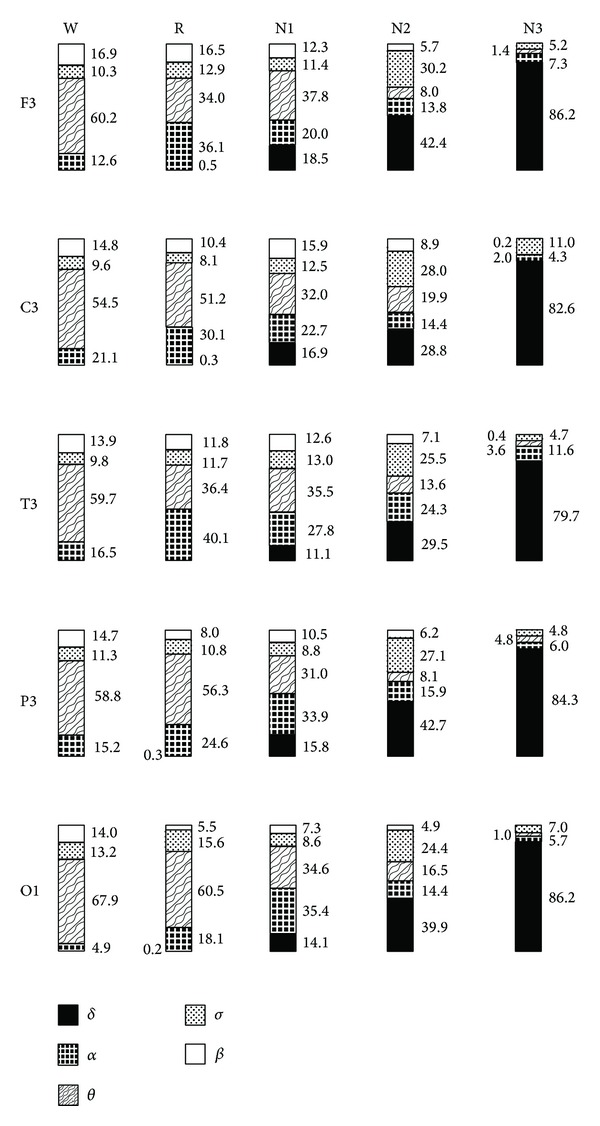
Spectral analysis of F3, C3, T3, P3, and O1 for all RISE episodes in the corresponding PTI channels (F3-F4, C3-C4, T3-T4, P3-P4, and O1-O2) compared to previous “baseline” EEG segments without elevated synchronization levels. Frequency bands: delta 0.5–3.5 Hz, theta > 3.5–8 Hz, alpha > 8–12 Hz, sigma > 12–14.5 Hz, and beta > 14.5–25 Hz. Although the mean levels and durations of synchronization during RISE episodes are largely independent of the current state of vigilance, the spectral content of EEG segments contributing to RISE changes extensively (the significance levels of the differences to the other stages are given in Table 3 for F3). See text for further details.

The EEG transients which caused an increase of synchronization could be identified visually in most cases. As expected, they depended largely on the respective state of vigilance and comprised the characteristic waveforms of each stage. The spectral analysis of the EEG transients confirmed these observations—their dominant frequency shift differed significantly for waking and sleep stages (Figure 3, Table 3). In waking, most events occurred in the alpha and to a lesser extent in the beta and theta range; the automatic spindle identification of the Brainlab system confirmed that the events with a dominant frequency shift in the sigma band during waking and REM sleep were not sleep spindles, but single waves or short wavetrains in the upper alpha and lower beta band. In NREM sleep, the alpha-predominance successively decreased, and delta increased markedly with the deepening of sleep. A large proportion of synchronized spindles was observed in N2 and to a lesser extent in N3. Alpha- and beta-dominant events in these stages were mostly correlated to arousals. In REM sleep, a larger proportion of theta events was found compared to waking and N1, while delta was nearly absent. In contrast to these changes with vigilance, regional differences were small (Figure 3), and hemispheric differences were absent.

## 4. Discussion

Research on EEG synchronization in sleep has mainly focused on changes of coupling levels and patterns during the sleep cycles and on correlating the different results to the waveforms that characterize, and in some cases define, the respective sleep stages [2, 5, 9–13]. Hence it was unexpected that our two previous studies on EEG coupling during activation phases in NREM sleep [15] and EEG synchronization in REM sleep [14] yielded very similar results as to the temporal and spatial distributions of synchronization patterns. Based on these findings, we extended the examination of EEG synchronization dynamics to the entire waking and sleep EEG. We were able to confirm that the fundamental synchronization pattern is independent of the state of vigilance, specific waveforms, and the spectral content of the EEG. For waking and all sleep stages, the durations of synchronized and unsynchronized EEG sections and their distributions were almost identical; all physiological waveforms contributed to the recurrent synchronizations. Synchronized and unsynchronized EEG sections were distinctly separated. The spatial distribution of mean synchronization levels was also constant with frontal and occipital maxima and central and midtemporal minima. Due to these results, we expand our proposition of a descriptive pattern of synchronization to all physiological states of vigilance: the recurrent increase of synchronization in the EEG, which we will address in the following as “RISE.”

### 4.1. Methods: PTI as a Measure of EEG Synchronization

EEG synchronization can be defined as the mutual adjustment of varying rhythms of coupled cortical oscillators; in this context it is regarded as a continuous dynamical process. The analysis of EEG synchronization patterns provides essential information about how interrelations between brain sites change with the wake-sleep cycle [5]. The coupling of different cortical sites can be measured by transinformation [19, 24], also referred to as mutual information [16, 18, 25]. EEG pointwise transinformation (PTI) provides insight into the spatiotemporal dynamics of these coupling patterns [19, 20]. We applied it successfully to the sleep EEG in two preliminary studies that focused on arousal-correlated activation phases in the NREM sleep EEG [15] and on recurrent increases of synchronization in the REM sleep EEG [14]. Some results of the first study were confirmed in another study on arousal-related decoupling of the sleep EEG [26]. Methodological issues of PTI as a measure of EEG synchronization are discussed in our previous studies [14, 15]. However, the issue of volume conduction has to be addressed here, since it can substantially influence the synchronization measures of adjacent EEG electrodes [27]. It is inevitable that volume conduction contributes to RISE when large potentials extend across a large area of the scalp; thus, not all increases of PTI are generated by active synchronization of cortical neuronal assemblies. On the other hand, our results point to a predominance of an active synchronization process, because the amount of synchronization measured by RISE is independent of the distance of the respective electrodes. In further contrast to our results, volume conduction would cause a parallel temporal dynamics and amplitude-dependent changes of PTI with EEG potentials. We can therefore conclude that volume conduction is not the main source of RISE, but it restricts the information RISE provides about the synchronization dynamics of cortical neuronal assemblies.

### 4.2. Results: Recurrent EEG Synchronization

Numerous synchronization measures have been applied to the physiological and pathological EEG [4, 11, 28–31]. Studies with conventional linear synchronization measures like ordinary and partial coherence and cross correlation [1, 2, 4–7, 32] reported spatial synchronization patterns that were reproduced by RISE: mean interhemispheric coupling levels were high in the anterior and posterior sites and low in the central and temporal sites. This regional distribution of synchronization also corresponds to the regional EEG slow-wave synchronization during sleep cyclic alternating pattern (CAP) A1 [33]. Since the time scales of the linear algorithms are much larger, a correspondence with the temporal properties of RISE was not to be expected. However, the algorithm and the physiological interpretation of PTI can be compared to the synchronization likelihood [34, 35]. The EEG slow-wave synchronization likelihood during normal sleep indicated higher spatial synchronization levels during CAP sleep in comparison to non-CAP sleep with fluctuations of the coupling level, probably corresponding to the single EEG slow waves [36]. This matches the moderate increase of mean synchronization levels in SWS in our study as well as the shorter intervals between synchronization phases; however, PTI is less affected by vigilance changes. This is somewhat unexpected, since PTI reacts to EEG synchrony variations on very short time scales and sensitively detects arousal-associated changes of EEG coupling [15, 26]. An obvious explanation is that the majority of EEG waves contributing to RISE are not related to arousals, so CAP and nCAP are not distinguished by EEG synchron. RISE is also independent of spectral power in contrast to CAP [36].

The median durations of PTI synchronization phases (350–450 ms) and unsynchronized intervals (1900–2200 ms except for SWS, see below) we obtained were comparable to the durations of stable microstates reported by two studies on spatiotemporal patterns of the alpha EEG [37, 38]. Similar dynamics in abrupt changes of microstates was also described. RISE was subtly modulated by SWS with increased synchronization levels and shorter unsynchronized segments in the frontal, central, and temporal regions and a higher frequency of synchronization phases in the anterior and midtemporal leads. This could be explained by the different spatial scales and cell population sizes associated with different EEG rhythms. The increase of synchronized intervals we observed in SWS might reflect the modulation of activity over larger spatial regions by low frequency oscillations, compared to the modulation in smaller spatial regions and shorter time windows by higher frequency oscillations [28, 39]. However, the increase of synchronization in SWS is more likely due to a methodological effect: when slow waves are synchronized by the same temporal pattern as other waves with higher frequencies, the longer duration of the slow waves causes a relative increase of mean PTI and a decrease of unsynchronized intervals in a given time interval. Indeed, the correction of the PTI parameters for this frequency-dependent effect by division by the relative amount of delta in the respective EEG channels removed the differences between SWS and the other stages.

### 4.3. Results: Spectral Analysis

In contrast to RISE, conventional spectral parameters vary extensively with the wake-sleep cycle [40–45]. Visual and spectral analysis confirmed that a multitude of waveforms and graphoelements contribute to RISE. The distribution of the dominant frequency shifts during increased synchronization in our study correlates well to the peaks of the power spectra of waking and sleep stages in previous studies [2, 46–49]. Moreover, the network organization of the EEG synchronization at different frequency bands during sleep might be a trait of all sleep stages [50], probably reflected by the stable pattern of RISE during sleep. This is especially evident for relatively stage-specific waveforms like delta waves and spindles. Likewise, the phasic EEG activation phases in NREM sleep induce short burst-like increases of EEG synchronization [15]. The cortical slow oscillation groups spindles, delta waves, and *K*-complexes and is itself synchronized in corresponding regions of both hemispheres [51]. Thus, the interplay of all these waveforms ought to establish the main framework of RISE during SWS.

Alpha waves contribute to RISE in different states of vigilance. Anterior and posterior alpha patterns of the characteristic waking-alpha rhythm are seemingly interconnected via corticocortical synapses and modulated thalamic inputs [3]; this could explain the large extension of synchronization peaks in RISE with waking-alpha activity as well as the results of EEG coherence studies [32, 52–54]. REM-specific alpha waves might be due to a distinct pattern of corticocortical coupling [3, 52], but they contribute to RISE the same way as alpha waves in waking and N1.

Beta and gamma oscillations have been examined preferentially in the context of cognitive processes [55]; these fast oscillations have been mostly attributed to active brain states in waking, REM sleep, and arousals from sleep [50, 51, 56, 57]. Although the traditional terminology views EEG epochs consisting mainly of fast activity as “desynchronized,” spontaneous fast activity is synchronized over cortical sites and through thalamocortical circuits [12]. Our results show a higher proportion of events in the beta range contributing to RISE during wakefulness, light sleep, and REM sleep than in SWS, in good agreement with the cited studies. In SWS, small amounts of fast activity play a part in RISE generation, too, probably during the depolarizing phase of the slow oscillation [12].

## 5. Conclusions

A dynamical, but qualitatively stable, pattern of recurrent EEG synchronization seems to continue throughout all physiological waking and sleep phases. RISE describes the rapid change of synchronized microstates with EEG waves across the whole spectrum as well as with state-specific EEG graphoelements, but it remains remarkably unaltered by changes of vigilance; thus, it seems to provide a persistent spatiotemporal framework for recurrent EEG synchronizations during the brain's resting state. The probable generators of RISE could be coupled corticocortical neuronal assemblies, modulated by thalamocortical and other subcorticocortical pathways; transient, but highly synchronized spatiotemporal configurations of these neuronal networks seem to alternate with phases of low synchronization. RISE needs to be evaluated for different pathological EEGs in future studies to examine its clinical relevance.

## Figures and Tables

**Table 1:** Essential sleep parameters of 21 patients.

	TST	W%	N1%	N2%	N3%	R%	SOL1	SOL2	REML
Mean	7.74 h	4.2	4.9	53.2	13.4	22.3	9.0 mn	14.8 mn	82.6 mn
Range	6.63–9.11 h	3.0–6.6	3.6–7.5	48.1–68.5	9.7–18.9	18.1–26.7	3.0–22.0 mn	4.5–27.0 mn	69.5–128.0 mn

Compilation of essential sleep parameters for all 21 normal sleepers who were included in the study. TST: total sleep time; W%–R%: percentage of respective stage; SOL1/2: sleep onset latency for N1/N2; REML: REM onset latency.

**Table 2:** Mean interhemispheric and transhemispheric pointwise transinformation (PTI) for waking and sleep stages.

	FP1-FP2	F3-F4	C3-C4	P3-P4	O1-O2	F7-F8	T3-T4	T5-T6	A1-A2	F3-O1	F4-O2
W_day_	0.271	0.163	0.104	0.111	0.208	0.098*	0.072*	0.193	0.220	0.164	0.157
W_night_	0.273	0.174	0.109	0.117	0.219	0.110*	0.068*	0.185	0.236	0.169	0.169
N1	0.320	0.203	0.139	0.142	0.250	0.123*	0.091*	0.205	0.312	0.197	0.176
N2	0.348	0.235	0.146	0.157	0.272	0.154	0.099	0.243	0.343	0.200	0.207
N3	0.563	0.406	0.202	0.232	0.402	0.251	0.192	0.372	0.455	0.350	0.369
R	0.249	0.144*	0.096	0.119	0.184	0.094*	0.066*	0.146*	0.217	0.117	0.126

Mean PTI of the waking phases during daytime (W_day_) and nighttime (W_night_) does not differ. Significant differences of mean PTI (∗) were found only for N3 versus W_day_, W_night_, N1, and R in the anterior and midtemporal channels and for N3 versus R also in the frontal and posterior temporal channels. This effect is probably caused by the large amount of delta waves in N3, which increase the duration of single synchronized episodes and, thus, the mean synchronization (see Section 4 for details).

**Table:** (a) Waking

	R	N1	N2	N3
Beta	—	—	0.05	0.001
Sigma	—	—	0.05	—
Alpha	—	—	0.01	0.001
Theta	0.05	—	—	—
Delta	—	0.001	0.001	0.001

**Table:** (b) REM sleep

	W	N1	N2	N3
Beta	—	—	0.05	0.001
Sigma	—	—	0.05	0.01
Alpha	—	—	0.01	0.05
Theta	0.05	—	0.05	0.01
Delta	—	0.01	0.001	0.001

**Table:** (c) N1 sleep

	W	R	N2	N3
Beta	—	—	0.05	0.001
Sigma	—	—	0.01	0.05
Alpha	—	—	0.01	0.001
Theta	—	—	—	0.05
Delta	0.001	0.01	0.05	0.01

**Table:** (d) N2 sleep

	W	R	N1	N3
Beta	0.05	0.05	0.05	0.001
Sigma	0.05	0.05	0.01	0.01
Alpha	0.01	0.01	0.01	0.01
Theta	—	—	—	—
Delta	0.001	0.001	0.05	0.05

**Table:** (e) N3 sleep

	W	R	N1	N2
Beta	0.001	0.001	0.001	0.001
Sigma	—	0.01	0.05	0.01
Alpha	0.001	0.05	0.001	0.01
Theta	—	0.01	0.05	—
Delta	0.001	0.001	0.01	0.05

“—”: not significant.

## Appendix

### A. Pointwise Transinformation

Time series of experimental measurements can be used to reconstruct the generating system in the phase space. For this purpose, vectors are constructed by delay coordinates with the embedding dimension *m* and the delay time *τ*. There are no unique criteria for the proper choice of values for *m* and *τ* under experimental conditions; however, a careful selection of these parameters is essential for an optimal reconstruction of relevant system trajectories [8, 58]. With adequate values for *m* and *τ*, the empirical point densities *p*_*i*_(*ε*) within hypercubes of the size *ε* around reference points determined by the time index *i* can be estimated (for further details see [14, 15]). We thus obtain the pointwise transinformation PTI [59]:

(A.1) $$\begin{array}{c} {\text{PTI}}_{i}\left(\varepsilon \right)=\log\frac{p_{i}^{\xi \chi }\left(\varepsilon \right)}{p_{i}^{\xi }\left(\varepsilon \right)p_{i}^{\chi }\left(\varepsilon \right)}, \end{array}$$

where *p*_*i*_^*ξ*^ and *p*_*i*_^*χ*^ are the probabilities for the occurrence of the values *ξ*_*i*_ and *χ*_*i*_ and *p*_*i*_^*ξχ*^(*ε*) is the probability for the occurrence of *χ*_*i*_ at the time of the occurrence of *ξ*_*i*_. For stochastic variables *ξ* and *χ*, PTI is 0, while PTI > 0 for any dependency between *ξ* and *χ*. For experimental data, PTI can reach negative values, so the dimension of PTI (“nats” or “bits”, depending on the base of the logarithm) was omitted here.

The probabilities *p*_*i*_^*ξ*^(*ε*), *p*_*i*_^*χ*^(*ε*), and *p*_*i*_^*ξχ*^(*ε*) were estimated by

(A.2) $$\begin{array}{c} p_{i}^{\xi }\left(\varepsilon \right)=\frac{1}{N}\overset{N}{\underset{j=1}{\sum }}\Theta \left(\varepsilon -\left|\xi_{i}-\xi_{j}\right|\right),\\ p_{i}^{\chi }\left(\varepsilon \right)=\frac{\text{1}}{N}\overset{N}{\underset{j=1}{\sum }}\Theta \left(\varepsilon -\left|\chi_{i}-\chi_{j}\right|\right),\\ p_{i}^{\xi \chi }\left(\varepsilon \right)=\frac{1}{N}\overset{N}{\underset{j=1}{\sum }}\Theta \left(\varepsilon -\left|\left(\xi_{i},\chi_{i}\right)-\left(\xi_{j},\chi_{j}\right)\right|\right). \end{array}$$

#### A.1. Choice of Parameter Values

For the choice of embedding parameter values, we used the pragmatic approach of our previous study [14]: in simulations on test signals and typical EEG graphoelements with different values for *m* and *τ*, we had found a minimum of *m* = 5 for an adequate representation of the coupling dynamics of all relevant EEG transients; a moderate increase of *m* improved the noise reduction and the discrimination of significant events. *m* = 8 was chosen for this study, which provided comparability to our previous results as well as a sufficient signal-noise ratio. With *τ* = 20 ms, changes on small time scales with frequencies up to 50 Hz can be registered. This choice of *τ* was also verified by the autocorrelation function, which approached 1/*e* for most screened EEG signals. We also confirmed, for all sleep stages of all EEGs, that in the range of *m* = 5 to 15 the quality of the results did not differ from the results we obtained with *m* = 8.

Like for *m* and *τ*, the choice of a value for *ε* is critical; obviously it must exceed the noise level and fall below the maximal amplitude of the analysed signal [60]. In the present study, the intervals with ∂PTI/∂ln⁡(*ε*) ≈ const. were calculated for all epochs of each EEG to register the relevant statistically dependent states of the respective generating system. A mean value of 31 *μ*V was found to be contained in all intervals with the condition ∂PTI/∂ln⁡(*ε*) ≈ const., so *ε* = 31 *μ*V was chosen for the further analyses.
